# Cyclo­hexa­nespiro-2′-[2′,3′,6′,7′-tetra­hydro-1′*H*-cyclo­penta­[*d*]pyrimidin]-4′(5′*H*)-one

**DOI:** 10.1107/S1600536809005388

**Published:** 2009-02-28

**Authors:** Daxin Shi, Dongfeng Qian, Qi Zhang, Jiarong Li

**Affiliations:** a School of Chemical Engineering and Environment, Beijing Institue of Technology, Beijing 100081, People’s Republic of China

## Abstract

The title compound, C_12_H_18_N_2_O, was synthesized by the reaction of cyclo­hexa­none and 2-amino­cyclo­pent-1-enecarbonitrile. In the mol­ecule of the title compound, the six-carbon ring displays a chair conformation, the six-membered 1,3-diaza ring and the cyclo­pentene ring both assume envelope conformations. Supra­molecular aggregation is achieved by N—H⋯O hydrogen bonds.

## Related literature

For general background on the biological activity of pyrimidinones, see: Schramm *et al.* (1984 ▶); Wen *et al.* (2002 ▶); For related structures, see: Yu *et al.* (1992 ▶); Zhang, Li, Shi *et al.* (2008 ▶); Zhang, Li, Yang *et al.* (2008 ▶).
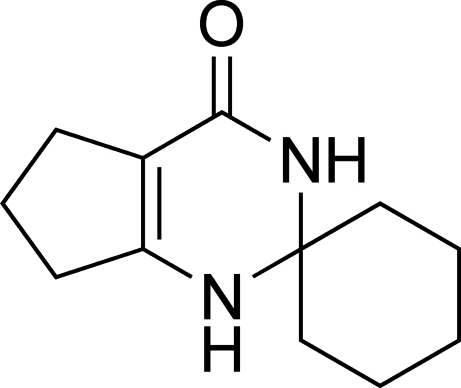


## Experimental

### 

#### Crystal data

C_12_H_18_N_2_O
*M* *_r_* = 206.28
Monoclinic, 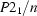

*a* = 10.294 (2) Å
*b* = 10.461 (2) Å
*c* = 10.659 (2) Å
β = 112.70 (3)°
*V* = 1059.0 (4) Å^3^
*Z* = 4
Mo *K*α radiation
μ = 0.08 mm^−1^
*T* = 113 K
0.24 × 0.20 × 0.08 mm

#### Data collection

Rigaku Saturn diffractometer
Absorption correction: multi-scan (*CrystalClear*; Rigaku/MSC, 2006 ▶) *T*_min_ = 0.980, *T*_max_ = 0.993
6976 measured reflections
1862 independent reflections
1632 reflections with *I* > 2σ(*I*)
*R*_int_ = 0.034

#### Refinement

*R*[*F* ^2^ > 2σ(*F* ^2^)] = 0.037
*wR*(*F* ^2^) = 0.103
*S* = 1.13
1862 reflections
144 parameters
2 restraints
H atoms treated by a mixture of independent and constrained refinement
Δρ_max_ = 0.22 e Å^−3^
Δρ_min_ = −0.22 e Å^−3^

### 

Data collection: *CrystalClear* (Rigaku/MSC, 2006 ▶); cell refinement: *CrystalClear*; data reduction: *CrystalClear*; program(s) used to solve structure: *SHELXS97* (Sheldrick, 2008 ▶); program(s) used to refine structure: *SHELXL97* (Sheldrick, 2008 ▶); molecular graphics: *SHELXTL* (Sheldrick, 2008 ▶); software used to prepare material for publication: *SHELXTL*.

## Supplementary Material

Crystal structure: contains datablocks global, I. DOI: 10.1107/S1600536809005388/pk2145sup1.cif

Structure factors: contains datablocks I. DOI: 10.1107/S1600536809005388/pk2145Isup2.hkl

Additional supplementary materials:  crystallographic information; 3D view; checkCIF report

## Figures and Tables

**Table 1:** Hydrogen-bond geometry (Å, °)

*D*—H⋯*A*	*D*—H	H⋯*A*	*D*⋯*A*	*D*—H⋯*A*
N1—H1⋯O1^i^	0.899 (9)	1.993 (9)	2.8832 (14)	169.9 (14)
N2—H2⋯O1^ii^	0.899 (9)	2.080 (11)	2.9458 (17)	161.3 (15)

Symmetry codes: (i) 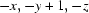; (ii) 
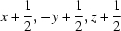
.

**Fig. 1.**
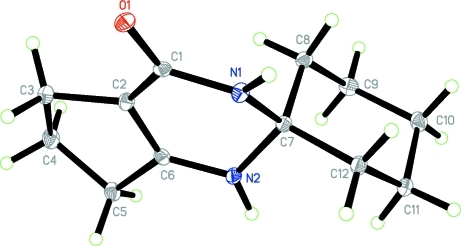
The molecular structure of the title compound, drawn with 30% probability ellipsoids.

**Fig. 2.**
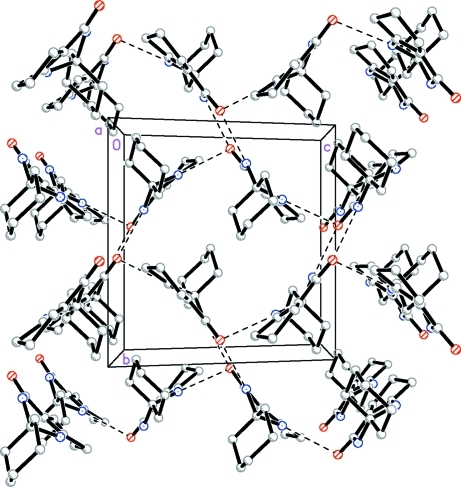
The crystal structure of the title compound, viewed along a axis.

## supplementary crystallographic information

### Comment

Pyrimidin-4(5*H*)-ones are valuable synthetic intermediates, and represent a common structure found in various biologically active compounds Schramm *et al.*, 1984). Functionalization of the pyrimidin-4(5*H*)-one group offers an attractive method for the generation of derivatives which may constitute interesting medicinal and biological properties. For example, spiro[cyclohexane-1,2'(1'H)-quinazolin]-4'(3'H)-one shows immunosuppressive, antifungal, and antitumor activity (Wen *et al.*, 2002).

Molecules of the title compound (Fig. 1) are linked by N1—H···O1 and N2—H···O1 H-bonds , as shown in Fig. 2 and have a similar conformation as (*s*)-2-(3-nitrophenyl)-1,2-dihydro-quinazolin-4(3*H*)-one (Zhang, Li & Shi *et al.*, 2008). The 1,3-diaza ring assumes envelope conformation, similar to that found in 4(3*H*)-quinazolinone derivatives (Zhang, Li & Yang *et al.*, 2008; Yu *et al.*, 1992). The cyclopentene exists in an envelope formation, and cyclohexane displays a chair conformation.

### Experimental

A solution of 2-aminocyclopent-1-enecarbonitrile (10 mmol, 1.08 g) and sodium methanolate (10 mmol, 0.54 g) in cyclohexanone (2 ml), was refluxed for 2 h. The reaction mixture was cooled to 293 K and kept at this temperature for an additional 12 h. The solvent was filtered *in vacuo* to give 2-cyclohexyl-2,3,6,7-tetrahydro-1*H*-cyclopenta[*d*]pyrimidin- 4(5*H*)-one. The product was recrystallizated from ethanol to give clearless crytals. *M*.p. 513–514 K; IR (KBr): 3201 (N—H), 3074, 2931 (C—H), 1707 (C=O) cm^-1^; ^1^H-NMR(DMSO, p.p.m.): 1.50 (6*H*, m), 1.74–1.81 (4*H*, m), 2.28 (2*H*, t), 2.38 (2*H*, t), 2.50 (2*H*, m), 6.57 (1*H*,s), 6.86 (1*H*, s). 50 mg of the product was dissolved in ethyl acetate (5 ml) and the solution was kept at room temperature for 4 days to give colorless single crystals.

### Refinement

H atoms attached to C were included in calculated positions with a riding model (C—H distance = 0.97 Å), while the N—H hydrogens were refined with N—H distance restraints of 0.90 Å. *U*_iso_ values were set to 1.2*U*_eq_ of the carrier atom.

### Crystal data

**Table:**

C_12_H_18_N_2_O	*F*(000) = 448
*M**_r_* = 206.28	*D*_x_ = 1.294 Mg m^−^^3^
Monoclinic, *P*2_1_/*n*	Mo *K*α radiation, λ = 0.71073 Å
Hall symbol: -P 2yn	Cell parameters from 3035 reflections
*a* = 10.294 (2) Å	θ = 2.8–27.8°
*b* = 10.461 (2) Å	µ = 0.08 mm^−^^1^
*c* = 10.659 (2) Å	*T* = 113 K
β = 112.70 (3)°	Plate, colorless
*V* = 1059.0 (4) Å^3^	0.24 × 0.20 × 0.08 mm
*Z* = 4	

### Data collection

**Table:**

Rigaku Saturn diffractometer	1862 independent reflections
Radiation source: rotating anode	1632 reflections with *I* > 2σ(*I*)
confocal multilayer optics	*R*_int_ = 0.034
Detector resolution: 14.63 pixels mm^-1^	θ_max_ = 25.0°, θ_min_ = 2.8°
ω scans	*h* = −12→12
Absorption correction: multi-scan (*CrystalClear*; Rigaku/MSC, 2006)	*k* = −12→12
*T*_min_ = 0.980, *T*_max_ = 0.993	*l* = −12→12
6976 measured reflections	

### Refinement

**Table:**

Refinement on *F*^2^	Primary atom site location: structure-invariant direct methods
Least-squares matrix: full	Secondary atom site location: difference Fourier map
*R*[*F*^2^ > 2σ(*F*^2^)] = 0.037	Hydrogen site location: inferred from neighbouring sites
*wR*(*F*^2^) = 0.103	H atoms treated by a mixture of independent and constrained refinement
*S* = 1.13	*w* = 1/[σ^2^(*F*_o_^2^) + (0.058*P*)^2^ + 0.1813*P*] where *P* = (*F*_o_^2^ + 2*F*_c_^2^)/3
1862 reflections	(Δ/σ)_max_ < 0.001
144 parameters	Δρ_max_ = 0.22 e Å^−^^3^
2 restraints	Δρ_min_ = −0.22 e Å^−^^3^

### Special details

**Table:**

Geometry. All e.s.d.'s (except the e.s.d. in the dihedral angle between two l.s. planes) are estimated using the full covariance matrix. The cell e.s.d.'s are taken into account individually in the estimation of e.s.d.'s in distances, angles and torsion angles; correlations between e.s.d.'s in cell parameters are only used when they are defined by crystal symmetry. An approximate (isotropic) treatment of cell e.s.d.'s is used for estimating e.s.d.'s involving l.s. planes.
Refinement. Refinement of *F*^2^ against ALL reflections. The weighted *R*-factor *wR* and goodness of fit *S* are based on *F*^2^, conventional *R*-factors *R* are based on *F*, with *F* set to zero for negative *F*^2^. The threshold expression of *F*^2^ > σ(*F*^2^) is used only for calculating *R*-factors(gt) *etc*. and is not relevant to the choice of reflections for refinement. *R*-factors based on *F*^2^ are statistically about twice as large as those based on *F*, and *R*- factors based on ALL data will be even larger.

### Fractional atomic coordinates and isotropic or equivalent isotropic displacement parameters (Å^2^)

**Table:**

	*x*	*y*	*z*	*U*_iso_*/*U*_eq_	
O1	−0.14346 (9)	0.42583 (8)	0.02403 (9)	0.0163 (2)	
N1	0.08172 (11)	0.34945 (10)	0.09960 (11)	0.0152 (3)	
N2	0.16234 (11)	0.18929 (10)	0.26816 (11)	0.0165 (3)	
C1	−0.05332 (13)	0.34458 (11)	0.09213 (13)	0.0137 (3)	
C2	−0.08039 (13)	0.24658 (11)	0.17362 (13)	0.0152 (3)	
C3	−0.21300 (13)	0.22403 (12)	0.19860 (14)	0.0179 (3)	
H3A	−0.2919	0.2044	0.1147	0.021*	
H3B	−0.2364	0.2976	0.2412	0.021*	
C4	−0.17249 (14)	0.10798 (13)	0.29541 (15)	0.0219 (3)	
H4A	−0.2158	0.1143	0.3614	0.026*	
H4B	−0.2031	0.0291	0.2447	0.026*	
C5	−0.01109 (13)	0.11111 (13)	0.36688 (14)	0.0196 (3)	
H5A	0.0185	0.1558	0.4531	0.023*	
H5B	0.0282	0.0255	0.3820	0.023*	
C6	0.03058 (13)	0.18298 (11)	0.26602 (13)	0.0152 (3)	
C7	0.17877 (12)	0.24061 (11)	0.14698 (13)	0.0142 (3)	
C8	0.14163 (13)	0.13822 (12)	0.03476 (14)	0.0165 (3)	
H8A	0.0486	0.1045	0.0181	0.020*	
H8B	0.1386	0.1776	−0.0487	0.020*	
C9	0.24726 (14)	0.02825 (12)	0.07214 (14)	0.0206 (3)	
H9A	0.2210	−0.0323	−0.0025	0.025*	
H9B	0.2451	−0.0159	0.1513	0.025*	
C10	0.39614 (13)	0.07713 (13)	0.10258 (15)	0.0212 (3)	
H10A	0.4621	0.0064	0.1303	0.025*	
H10B	0.4007	0.1147	0.0212	0.025*	
C11	0.43647 (13)	0.17692 (13)	0.21547 (14)	0.0194 (3)	
H11A	0.5287	0.2112	0.2298	0.023*	
H11B	0.4422	0.1364	0.2993	0.023*	
C12	0.32970 (12)	0.28661 (12)	0.18128 (14)	0.0171 (3)	
H12A	0.3561	0.3445	0.2582	0.021*	
H12B	0.3333	0.3340	0.1045	0.021*	
H1	0.1032 (15)	0.4130 (12)	0.0541 (14)	0.025 (4)*	
H2	0.2347 (13)	0.1526 (15)	0.3359 (14)	0.034 (5)*	

### Atomic displacement parameters (Å^2^)

**Table:**

	*U*^11^	*U*^22^	*U*^33^	*U*^12^	*U*^13^	*U*^23^
O1	0.0151 (5)	0.0148 (4)	0.0169 (5)	0.0014 (4)	0.0038 (4)	0.0029 (4)
N1	0.0145 (6)	0.0128 (5)	0.0185 (6)	−0.0001 (4)	0.0066 (5)	0.0035 (5)
N2	0.0131 (6)	0.0219 (6)	0.0142 (6)	0.0028 (4)	0.0050 (5)	0.0048 (5)
C1	0.0142 (6)	0.0128 (6)	0.0121 (7)	−0.0016 (5)	0.0030 (5)	−0.0031 (5)
C2	0.0147 (6)	0.0144 (6)	0.0171 (7)	−0.0002 (5)	0.0067 (5)	0.0001 (5)
C3	0.0161 (6)	0.0178 (6)	0.0211 (7)	−0.0003 (5)	0.0087 (5)	0.0006 (6)
C4	0.0209 (7)	0.0242 (7)	0.0239 (8)	−0.0002 (6)	0.0125 (6)	0.0058 (6)
C5	0.0210 (7)	0.0214 (7)	0.0188 (8)	0.0034 (6)	0.0105 (6)	0.0054 (6)
C6	0.0178 (7)	0.0140 (6)	0.0152 (7)	−0.0009 (5)	0.0080 (5)	−0.0017 (5)
C7	0.0134 (6)	0.0146 (6)	0.0144 (7)	0.0003 (5)	0.0052 (5)	0.0021 (5)
C8	0.0161 (7)	0.0167 (6)	0.0157 (7)	−0.0015 (5)	0.0052 (5)	−0.0003 (5)
C9	0.0243 (7)	0.0160 (6)	0.0214 (8)	0.0009 (5)	0.0087 (6)	−0.0021 (5)
C10	0.0208 (7)	0.0234 (7)	0.0219 (8)	0.0052 (6)	0.0110 (6)	0.0003 (6)
C11	0.0134 (6)	0.0256 (7)	0.0197 (7)	0.0017 (5)	0.0068 (5)	−0.0002 (6)
C12	0.0151 (7)	0.0182 (6)	0.0190 (7)	−0.0021 (5)	0.0076 (5)	−0.0026 (5)

### Geometric parameters (Å, °)

**Table:**

O1—C1	1.2612 (15)	C5—H5B	0.9700
N1—C1	1.3623 (16)	C7—C12	1.5299 (17)
N1—C7	1.4698 (16)	C7—C8	1.5399 (18)
N1—H1	0.899 (9)	C8—C9	1.5265 (18)
N2—C6	1.3494 (17)	C8—H8A	0.9700
N2—C7	1.4676 (17)	C8—H8B	0.9700
N2—H2	0.899 (9)	C9—C10	1.5271 (19)
C1—C2	1.4387 (17)	C9—H9A	0.9700
C2—C6	1.3599 (18)	C9—H9B	0.9700
C2—C3	1.5068 (17)	C10—C11	1.5246 (19)
C3—C4	1.5429 (19)	C10—H10A	0.9700
C3—H3A	0.9700	C10—H10B	0.9700
C3—H3B	0.9700	C11—C12	1.5324 (18)
C4—C5	1.5380 (19)	C11—H11A	0.9700
C4—H4A	0.9700	C11—H11B	0.9700
C4—H4B	0.9700	C12—H12A	0.9700
C5—C6	1.5036 (18)	C12—H12B	0.9700
C5—H5A	0.9700		
			
C1—N1—C7	122.29 (10)	N1—C7—C12	109.36 (10)
C1—N1—H1	117.0 (9)	N2—C7—C8	110.53 (10)
C7—N1—H1	118.8 (9)	N1—C7—C8	109.86 (10)
C6—N2—C7	117.35 (11)	C12—C7—C8	109.22 (10)
C6—N2—H2	120.6 (11)	C9—C8—C7	112.53 (11)
C7—N2—H2	121.4 (11)	C9—C8—H8A	109.1
O1—C1—N1	121.07 (11)	C7—C8—H8A	109.1
O1—C1—C2	123.83 (11)	C9—C8—H8B	109.1
N1—C1—C2	114.98 (11)	C7—C8—H8B	109.1
C6—C2—C1	118.79 (11)	H8A—C8—H8B	107.8
C6—C2—C3	111.14 (11)	C8—C9—C10	111.01 (11)
C1—C2—C3	128.16 (11)	C8—C9—H9A	109.4
C2—C3—C4	102.23 (10)	C10—C9—H9A	109.4
C2—C3—H3A	111.3	C8—C9—H9B	109.4
C4—C3—H3A	111.3	C10—C9—H9B	109.4
C2—C3—H3B	111.3	H9A—C9—H9B	108.0
C4—C3—H3B	111.3	C11—C10—C9	110.02 (11)
H3A—C3—H3B	109.2	C11—C10—H10A	109.7
C5—C4—C3	106.03 (10)	C9—C10—H10A	109.7
C5—C4—H4A	110.5	C11—C10—H10B	109.7
C3—C4—H4A	110.5	C9—C10—H10B	109.7
C5—C4—H4B	110.5	H10A—C10—H10B	108.2
C3—C4—H4B	110.5	C10—C11—C12	111.91 (11)
H4A—C4—H4B	108.7	C10—C11—H11A	109.2
C6—C5—C4	102.01 (11)	C12—C11—H11A	109.2
C6—C5—H5A	111.4	C10—C11—H11B	109.2
C4—C5—H5A	111.4	C12—C11—H11B	109.2
C6—C5—H5B	111.4	H11A—C11—H11B	107.9
C4—C5—H5B	111.4	C7—C12—C11	112.98 (10)
H5A—C5—H5B	109.2	C7—C12—H12A	109.0
N2—C6—C2	123.11 (12)	C11—C12—H12A	109.0
N2—C6—C5	125.07 (11)	C7—C12—H12B	109.0
C2—C6—C5	111.77 (11)	C11—C12—H12B	109.0
N2—C7—N1	106.97 (10)	H12A—C12—H12B	107.8
N2—C7—C12	110.86 (11)		
			
C7—N1—C1—O1	−163.93 (11)	C4—C5—C6—C2	16.59 (14)
C7—N1—C1—C2	19.87 (17)	C6—N2—C7—N1	40.32 (14)
O1—C1—C2—C6	−165.34 (12)	C6—N2—C7—C12	159.48 (11)
N1—C1—C2—C6	10.73 (17)	C6—N2—C7—C8	−79.26 (13)
O1—C1—C2—C3	−2.6 (2)	C1—N1—C7—N2	−44.20 (15)
N1—C1—C2—C3	173.52 (12)	C1—N1—C7—C12	−164.32 (11)
C6—C2—C3—C4	−15.01 (14)	C1—N1—C7—C8	75.81 (14)
C1—C2—C3—C4	−178.87 (13)	N2—C7—C8—C9	−67.76 (13)
C2—C3—C4—C5	24.67 (14)	N1—C7—C8—C9	174.42 (10)
C3—C4—C5—C6	−25.13 (14)	C12—C7—C8—C9	54.46 (14)
C7—N2—C6—C2	−15.31 (18)	C7—C8—C9—C10	−57.49 (15)
C7—N2—C6—C5	167.51 (12)	C8—C9—C10—C11	56.57 (15)
C1—C2—C6—N2	−12.96 (19)	C9—C10—C11—C12	−55.28 (15)
C3—C2—C6—N2	−178.51 (11)	N2—C7—C12—C11	69.13 (14)
C1—C2—C6—C5	164.56 (11)	N1—C7—C12—C11	−173.17 (11)
C3—C2—C6—C5	−1.00 (15)	C8—C7—C12—C11	−52.91 (15)
C4—C5—C6—N2	−165.96 (12)	C10—C11—C12—C7	54.85 (15)

### Hydrogen-bond geometry (Å, °)

**Table:**

*D*—H···*A*	*D*—H	H···*A*	*D*···*A*	*D*—H···*A*
N1—H1···O1^i^	0.90 (1)	1.99 (1)	2.8832 (14)	170 (1)
N2—H2···O1^ii^	0.90 (1)	2.08 (1)	2.9458 (17)	161 (2)

Symmetry codes: (i) −*x*, −*y*+1, −*z*; (ii) *x*+1/2, −*y*+1/2, *z*+1/2.
